# Clinical features and risk factors for severe inpatients with COVID-19: A retrospective study in China

**DOI:** 10.1371/journal.pone.0244125

**Published:** 2020-12-17

**Authors:** Juan Wang, Shuzhen Guo, Yili Zhang, Kuo Gao, Jiacheng Zuo, Nannan Tan, Kangjia Du, Yan Ma, Yong Hou, Quntang Li, Hongming Xu, Jin Huang, Qiuhua Huang, Hui Na, Jingwei Wang, Xiaoyan Wang, Yanhua Xiao, Junteng Zhu, Hong Chen, Zhang Liu, Mingxuan Wang, Linsong Zhang, Wei Wang

**Affiliations:** 1 Department of Education Administration, Beijing University of Chinese Medicine, Beijing, China; 2 Department of Scientific Research, Beijing University of Chinese Medicine, Beijing, China; 3 School of Traditional Chinese Medicine, Beijing University of Chinese Medicine, Beijing, China; 4 Institute of Basic Research in Clinical Medicine, China Academy of Chinese Medical Sciences, Beijing, China; 5 The First Affiliated Hospital of Anhui University of Traditional Chinese Medicine, Hefei, Anhui, China; 6 Chongqing Traditional Chinese Medicine Hospital, Chongqing, China; 7 Department of Infectious Disease, Daqing Second Hospital, Daqing, Heilongjiang, China; 8 Department of Traditional Chinese Medicine, The People’s Hospital of GuangXi Zhuang Autonomous Region, Nanning, Guangxi, China; 9 Department of Infectious Disease, Harbin Infectious Disease Hospital, Harbin, Heilongjiang, China; 10 Department of Infectious Disease, Jinzhong Infectious Disease Hospital, Jinzhong, Shanxi, China; 11 Department of Traditional Chinese Medicine, Mudanjiang Kangan Hospital, Mudanjiang, Heilongjiang, China; 12 Department of Rehabilitation Medicine, The Affiliated Hospital of Putian College, Putian, Fujian, China; 13 President’s Office, The First Hospital of Qiqihar, Qiqihar, Heilongjiang, China; 14 Department of Traditional Chinese Medicine, The First Hospital of Suihua City, Suihua, Heilongjiang, China; 15 Department of Traditional Chinese Medicine, Suining Central Hospital, Suining, Sichuan, China; 16 Department of Traditional Chinese Medicine, Hospital (T·C·M) Affiliated to Southwest Medical University, Luzhou, Sichuan, China; 17 President’s Office, Beijing University of Chinese Medicine, Beijing, China; University Magna Graecia of Catanzaro, ITALY

## Abstract

**Background:**

A worldwide outbreak of coronavirus disease (COVID-19), since 2019, has brought a disaster to people all over the world. Many researchers carried out clinical epidemiological studies on patients with COVID-19 previously, but risk factors for patients with different levels of severity are still unclear.

**Methods:**

562 patients with laboratory-confirmed COVID-19 from 12 hospitals in China were included in this retrospective study. Related clinical information, therapies, and imaging data were extracted from electronic medical records and compared between patients with severe and non-severe status. We explored the risk factors associated with different severity of COVID-19 patients by logistic regression methods.

**Results:**

Based on the guideline we cited, 509 patients were classified as non-severe and 53 were severe. The age range of whom was 5–87 years, with a median age of 47 (IQR 35.0–57.0). And the elderly patients (older than 60 years old) in non-severe group were more likely to suffer from fever and asthma, accompanied by higher level of D-dimer, red blood cell distribution width and low-density lipoprotein. Furthermore, we found that the liver and kidney function of male patients was worse than that of female patients in both severe and non-severe groups with different age levels, while the severe females had faster ESR and lower inflammatory markers. Of major laboratory markers in non-severe cases, baseline albumin and the lymphocyte percentage were higher, while the white blood cell and the neutrophil count were lower. In addition, severe patients were more likely to be accompanied by an increase in cystatin C, mean hemoglobin level and a decrease in oxygen saturation. Besides that, advanced age and indicators such as count of white blood cell, glucose were proved to be the most common risk factors preventing COVID-19 patients from aggravating.

**Conclusion:**

The potential risk factors found in our study have shown great significance to prevent COVID-19 patients from aggravating and turning to critical cases during treatment. Meanwhile, focusing on gender and age factors in groups with different severity of COVID-19, and paying more attention to specific clinical symptoms and characteristics, could improve efficacy of personalized intervention to treat COVID-19 effectively.

## Introduction

Since December 2019, the Corona Virus Disease 2019 (COVID-19) has become the latest global health threat [1]. The pathogen has typical coronoviridae characteristics [2]. On January 30, 2020, WHO officially declared the COVID-19 epidemic as a Public Health Emergency of International Concern [3]. Currently, the emerging virus rapidly became a challenge for global public health due to spread by human-to-human transmission [4]. As of Oct 13, 2020, more than 37,801,526 cumulative confirmed cases of COVID-19 have been reported in 189 countries or regions, with more than 1,080,680 death [5].

Judging from the existing evidence, most COVID-19 patients were classified as mild (81%), ordinary (14%), severe and critical (5%) [6, 7]. The clinical characteristics of patients with COVID-19 were described and the differences between mild and severe patients were compared in Previous studies [8, 9]. However, some patients, especially with elderly and previous co-existing diseases, may develop serious diseases and die of multiple organ failure in a short period of time [10]. Thus, the effective indicators to evaluate the severity and clinical progress of the disease still need to be further studied, which is undoubtedly helpful to reduce the mortality of COVID-19. Although many risk factors such as advanced age, underlying chronic disease and low immune function have been shown to predict the prognosis of patients infected with SARS-CoV-2, the risk factors for the severity of the disease are not clear [11, 12].

Previous study has showed that the incidence risk of COVID-19 might be as low as 0.1 for children, while it could be over 0.9 for middle aged adults. The mortality risk might be above 0.2 for patients older than 80 years. Severe COVID-19 was also more common in males than females [13]. However, information regarding the prevalence and recovery rate for clinical features and epidemiology of COVID-19 for different population remains scarce.

In present study, therefore, we collected 562 COVID-19 cases from 12 hospitals in China from January 28 to February 25, 2020. On the basis of a comprehensive description of the clinical characteristics of above cases, we focused on different genders and age factors in severe and non-severe groups. In addition, we tried to explore potential risk factors of COVID-19 patients with different severity. Also, the goal of the current study was to provide references for early detection and treatment timely to prevent non-severe COVID-19 patients from poor prognosis.

## Materials and methods

### Study design and participants

This was a retrospective case study that included 562 patients with COVID -19 from 12 hospitals in 7 provinces (Anhui, Chongqing, Heilongjiang, Shanxi, Sichuan, Fujian and Guangxi) of China (S1 Table) under the direction of the National Health Commission, from January 28 to February 25, 2020. All patients were diagnosed with COVID-19 according to the criteria: (1) Epidemiology history, (2) Fever (defined as axillary temperature of at least 37.3°C) or other respiratory symptoms, (3) Typical CT image abnormities of viral pneumonia, and (4) Positive result of RT-PCR for SARS-CoV-2 RNA. This study was approved by State Administration of Traditional Chinese Medicine, Administration of Traditional Chinese Medicine of the above provinces and the institutional board of 12 participating setting. Due to the emerging infectious diseases, the written informed consent was waived.

### Data collection

We extracted demographic data, medical history, exposure history, symptoms and signs, laboratory findings and imaging data from electronic medical records. The date of onset was defined as the date on which symptoms occur for the first time. All data were analyzed by the research team and double checked by two physicians and a third researcher adjudicated any difference in interpretation between the two primary reviewers.

### Laboratory procedures

Methods for laboratory confirmation of SARS-CoV-2 infection have been described in previous studies [14]. Briefly, twelve institutions were responsible for SARS-CoV-2 detection in respiratory specimens by next-generation sequencing or real-time RT-PCR methods. Throat-swab specimens were obtained for SARS-CoV-2 PCR re-examination every other day after clinical remission of symptoms. The criteria for discharge were absence of fever for at least 3 days, substantial improvement of the chest CT report in both lungs, clinical remission of respiratory symptoms, and two throat-swab samples negative for SARS-CoV-2 RNA obtained at least 24h apart. Routine blood examinations included blood cell series, white blood cell series, platelet series, biochemical tests, myocardial enzymes and procalcitonin. Furthermore, CT imaging features were used to quantify the pathological changes of COVID-19 patients.

### Definitions

The severity of COVID-19 was determined according to the Chinese COVID-19 Management guidelines (version 6.0) [15]. Patients with mild clinical symptoms and no pneumonia manifestation found in imaging were defined as mild cases. Ordinary cases were diagnosed as patients had symptoms such as fever and respiratory tract symptoms, etc. and pneumonia manifestation could be seen in imaging. Severe cases were diagnosed as meeting any of the following: 1. Breathing difficulty, RR ≥30 breaths/min; 2. Pulse oxygen saturation (SpO_2_) ≤ 93% on room air at rest state; 3. Arterial partial pressure of oxygen (PaO_2_)/oxygen concentration (FiO_2_) ≤ 300 mmHg (1 mmHg = 0.133 kPa). For high altitude areas (above 1 kilometer), PaO2/FiO_2_ values should be adjusted based on equation of PaO2/FiO2×[Atmospheric Pressure (mmHg)/760]. Patients with >50% lesions progression within 24 to 48 hours in pulmonary imaging should be treated as severe cases. Critical case was diagnosed as meeting any of the following: 1. Respiratory failure and mechanical ventilation needed; 2. Shock occurs; 3. Multiple organ failure and monitoring and treatment in ICU required [16]. In our study, we divided all patients into severe cases (including severe and critical cases) and non-severe cases (including mild and ordinary cases) for further analysis. Hypertension, diabetes, coronary heart disease and other comorbidity information were recorded according to their medical history reported.

### Statistical analysis

Categorical variables were represented by frequency and percentage (%). If the continuous variable had a non-normal distribution, the median (IQR) would be used. The χ2 test or Fisher exact test were used to compare categorical variables between groups, while Students t test or Mann–Whitney U test were applied to continuous variables analysis as appropriate [17]. In addition, we conducted an in-depth analysis of the data from the dimensions of age and gender in both severe and non-severe groups. Univariate and multivariate logistic regression models were used to explore the risk factors related to the severity of COVID-19 patients. Considering the total number of severe cases (n = 53) in our study and to avoid overfitting in the model, five variables were chosen for multivariable analysis based on previous findings. Previous studies have shown white blood cell to be higher in critically ill cases, whereas symptoms including fever and asthma have been less commonly observed in non-severe patients [8, 18, 19]. Similar risk factors, including older age, have been associated with adverse clinical outcomes in adults with COVID-19 [20]. Besides that, glucose was chosen as the fifth variable for our multivariable logistic regression model. We excluded variables from the univariable analysis if their accuracy was unconfirmed (e.g. exposure, which was self-reported), or if missing value was too limited to calculate odds ratios. We compared clinical data from multiple dimensions, and a generalized linear model was used to adjust for possible differences in patients’ characteristics between two groups. SPSS software (version 21.0) and SAS software (version 9.4) were applied for all statistical analysis, while the *p*-value < 0.05 was considered significant in our study.

## Results

Of 562 hospitalized patients with COVID-19 from 12 hospitals in 7 provinces in China (S1 Table), the median age was 47 years (IQR 35.0–57.0), ranging from 5 years to 87 years, and most patients were male (Table 1). We also found the median age of severe group was older than non-severe group (Table 1).

**Table 1:** Demographic, clinical, laboratory, and radiographic findings of patients on admission.

	Total	Non-severe	Severe	p value
	(n = 562)	(n = 509)	(n = 53)	
**Demographics**				
Age, years	47.00((35.00–57.00)	46.00(33.00–56.00)	59.00(48.00–65.00)	<0.0001
Epidemic area exposure history	171(30.42%)	164(32.22%)	7(13.20%)	0.004
**Symptoms and signs**				
Fever	226(40.21%)	191(37.52%)	35(66.04%)	<0.0001
Fatigue	107(19.04%)	87(17.09%)	20(37.74%)	<0.0001
Cough	287(51.07%)	245(48.13%)	42(79.25%)	<0.0001
Anorexia	82(14.59%)	69(13.56%)	13(24.53%)	0.031
Diarrhea	59(10.50%)	58(11.39%)	1(1.89%)	0.032
Asthma	68(12.10%)	46(9.04%)	22(41.51%)	<0.0001
**Laboratory findings**				
Albumin, g/L	39.70(35.88–43.93)	39.95(36.00–44.00)	37.97(33.93–42.56)	0.037
Cystatin C, mg/L				
<0.54	2(0.36%)	2(0.39%)	0	0.017
0.54–1.5	182(32.38%)	165(32.42%)	17(32.08%)	..
>1.5	11(1.96%)	7(1.38%)	4(7.55%)	..
White blood cell count, 10^9/L	5.86(4.49–7.56)	5.80(4.46–7.47)	6.65(4.61–9.30)	0.043
<3.5	45(8.01%)	43(8.45%)	2(3.77%)	0.015
3.5–9.5	402(71.53%)	368(72.30%)	34(64.15%)	..
>9.5	50(8.90%)	40(7.86%)	10(18.87%)	..
Neutrophil count, 10^9/L	3.70(2.74–5.32)	3.62(2.70–5.20)	4.86(3.17–7.69)	0.013
<1.8	36(6.41%)	34(6.68%)	2(3.77%)	0.013
1.8–6.3	381(67.79%)	352(69.16%)	29(54.72%)	..
>6.3	79(14.06%)	65(12.77%)	14(26.42%)	..
Lymphocyte percentage, %	24.30(16.98–32.70)	24.65(17.88–33.00)	20.90(10.40–29.18)	0.010
Mean hemoglobin, pg				
<27	47(8.36%)	45(8.84%)	2(3.77%)	0.051
27–34	441(78.47%)	399(78.39%)	42(79.25%)	..
>34	16(2.85%)	12(2.36%)	4(7.55%)	..
Oxygen saturation, %				
<92.5	4(0.71%)	3(0.59%)	1(1.89%)	0.053
92.5~98.5	51(9.07%)	49(9.63%)	2(3.77%)	..
>98.5	24(4.27%)	24(4.72%)	0	..

Data are median (IQR), n (%), or n/N (%). p values were calculated by Mann-Whitney U test, χ^2^ test, or Fisher’s exact test, as appropriate.

Hypertension was reported as the most common comorbidity among these patients, followed by diabetes and heart diseases (Table 1 and S2 Table). Fever and cough were the most common symptoms on admission (Table 1 and S3 Table). Major laboratory markers were tracked from illness onset (Table 1). Of major above laboratory markers, baseline albumin and the lymphocyte percentage were higher, whereas, non-severe patients were accompanied by lower white blood cell count and neutrophil count (Table 1). There were significant differences in cystatin C, white blood cell count, neutrophil count, average hemoglobin and blood oxygen saturation. Two-side ground-glass opacity was most commonly imaging features between two groups (Table 1).

Furthermore, we made a detailed comparison of male and female patients in each group (Table 2). In non-severe group, female patients were more prone to have fatigue and anorexia symptoms than male, and more use of antiviral drugs. Besides that, erythrocyte sedimentation rate of female patients was faster than males’, and the inflammation index including white blood cells, neutrophils decreased more significantly. However, male patients had worse liver and kidney function than female cases in indirect bilirubin, creatinine, uric acid, cystatin and glomerular filtration rate (Table 2). And in severe cases, females were more prone to have cough and the recovery rate was lower. It seems that male patients have more obvious symptoms of infection, of which the level of high hypersensitivity C-reactive protein and creatinine increased (Table 2). We also made a comparison of patients with different age in both non-severe and severe cases. The results showed that the elderly non-severe patients were more prone to have fever, cough, and asthma, and the cure rate of which was also the lowest. They tended to have the highest low-density lipoprotein, red blood cell volume distribution width and D-dimer. Hypertension was more obvious in these patients. And in severe cases, the median hemoglobin concentration of patients over 60 years was lowest (Table 3).

**Table 2:** Clinical characteristics and laboratory findings of male and female patients in different illness severity.

**Non-severe**				
	**Total**	**Male**	**Female**	**P value**
	(n = 509)	(n = 265)	(n = 244)	
**Symptoms and signs**				
Fatigue	87(17.09%)	35(13.21%)	52(21.31%)	0.015
Anorexia	69(13.56%)	28(10.57%)	41(16.80%)	0.04
**Laboratory findings**				
γ-glutamyl transpeptidase, IU/L	30.27(19.00–57.09)	34.70(20.39–58.00)	26.00(17.00–51.00)	0.006
Indirect bilirubin, umol/L	7.50(4.80–11.23)	7.70(5.39–11.60)	6.90(3.93–10.00)	0.041
Uric acid, umol/L	278.97(218.00–348.11)	295.00(230.02–368.30)	269.70(206.00–336.00)	0.029
Creatinine, umol/L	61.66(51.63–75.00)	64.60(54.94–76.70)	59.80(49.00–70.60)	<0.0001
Cystatin C, mg/L	0.935(0.8–1.1)	1.00(0.81–1.17)	0.90(0.78–1.04)	0.009
Glomerular filtration rate	110.82(102.37–122.09)	108.28(99.31–114.99)	115.70(103.74–127.98)	0.024
Haemoglobin, g/L	134.00(123.00–147.50)	137.00(124.00–148.00)	131.00(119.50–147.00)	0.026
Mean hemoglobin concentration, g/L	335.00(326.00–344.00)	336.50(327.00–345.00)	333.00(325.00–342.00)	0.016
Erythrocyte sedimentation rate, MM/h	24.00(10.00–38.00)	20.00(9.00–30.00)	30.00(10.75–49.25)	0.016
α-hydroxybutyrate dehydrogenase 72–182, IU/L	143.00(126.00–161.00)	153.00(133.00–169.00)	136.50(122.89–152.26)	0.039
Total bilirubin1.7–20, umol/L	10.33(6.77–13.82)	10.68(7.30–14.50)	10.10(6.10–13.30)	0.038
Direct bilirubin<15, umol/L	6.80(4.30–9.27)	7.40(4.90–10.20)	6.00(3.50–8.68)	<0.0001
Cystatin C 0.54–1.5, mg/L	0.91(0.80–1.09)	0.98(0.81–1.13)	0.89(0.78–1.01)	0.008
Glomerular filtration rate>90	111.93(105.09–123.00)	109.16(103.69–116.76)	116.40(106.47–128.79)	0.038
White blood cell count<3.5, 10^9/L	3.08(2.51–3.24)	3.22(3.06–3.36)	2.73(2.38–3.16)	0.008
Neutrophil count<1.8, 10^9/L	1.47(1.18–1.70)	1.70(1.52–1.76)	1.29(1.09–1.46)	<0.0001
Haemoglobin115-150, g/L	133.00(126.00–142.00)	135.00(127.00–143.00)	131.00(125.00–138.00)	0.025
Red blood cell distribution width SD<39, fL	37.30(36.00–38.03)	37.00(35.90–37.90)	37.50(36.40–38.20)	0.033
Erythrocyte sedimentation rate >20, MM/h	35.00(27.50–52.50)	30.00(24.75–45.50)	39.00(31.29–59.00)	0.005
γ-glutamyl transpeptidase7-45, IU/L	22.00(16.00–30.00)	23.00(18.00–31.20)	20.96(15.00–27.00)	0.009
**Severe**				
	**Total**	**Male**	**Female**	P value
	**(n = 49, deletion in 4 cases)**	**(n = 21, deletion in 4 cases)**	**(n = 28)**	
**Demographics**				
Age	59(49–65)	54(44–62)	62(56–71.75)	0.009
**Symptoms and signs**				
Cough	6(11.32%)	6(24%)	0	0.006
**Comorbidity**				
Hypertension	42(79.25%)	16(64%)	26(92.86%)	0.01
**Laboratory findings**				
Total bile acid,umol/L	3.30(2.10–4.20)	2.75(1.95–3.55)	3.70(2.90–4.70)	0.023
Amylase,U/L	58.00(38.00–78.83)	66.00(62.00–82.20)	49.00(36.00–78.33)	0.029
Creatinineu,mol/L	67.20(54.85–78.25)	72.20(58.75–87.92)	60.70(52.05–74.27)	0.037
Basophil count,10^9/L	0.01(0.01–0.03)	0.02(0.01–0.04)	0.01(0–0.02)	0.035
Hypersensitive C-reactive protein, mg/L	2.89(1.21–8.01)	8.01(2.48–19.35)	1.61(0.92–3.24)	0.028
total bile acid<20, umol/L	3.30(2.10–4.20)	2.75(1.95–3.55)	3.70(2.90–4.70)	0.023
Platelet count 125–350,10^9/L	207.00(156.00–27.00)	233.00(185.50–287.00)	171.00(134.00–258.00)	0.043
C-Reactive Protein<10, mg/L	4.72(1.58–6.28)	1.81(1.40–4.05)	6.10(3.30–8.03)	0.05
Hypersensitive C-Reactive Protein>0.5,mg/L	2.89(1.21–8.01)	8.01(2.48–19.35)	1.61(0.92–3.24)	0.028
Fibrinogen1.7~4, g/L	2.85(2.22–3.49)	3.31(2.82–3.69)	2.20(1.98–2.88)	0.009

**Table 3:** Clinical characteristics and laboratory findings of three age groups in different illness severity.

**Non-severe**					
	**Total**	Youth (<30 yr)	Middle age (30–60 yr)	Senior (> 60 yr)	P value
	(n = 509)	(n = 101)	(n = 327)	(n = 81)	
**Comorbidity**					
Hypertension	78(15.32%)	22(21.78%)	51(15.60%)	5(6.17%)	0.014
**Symptoms and signs**					
Fever	191(37.52%)	27(26.73%)	127(38.84%)	37(45.68%)	0.023
Asthma	46(9.04%)	2(1.98%)	33(10.09%)	11(13.58%)	0.014
Cough	245(48.13%)	35(34.65%)	167(51.07%)	43(53.09%)	0.01
**Laboratory findings**					
Low-Density Lipoprotein, mmol/L	2.43(1.96–3.00)	2.16(1.91–2.56)	2.48(1.94–2.98)	2.74(2.22–3.30)	0.045
Red Blood Cell Distribution Width Cv,%	12.50(12.00–13.30)	12.70(12.00–13.75)	12.40(12.00–13.10)	12.85(12.00–13.70)	0.023
D-dimer, μg/Ml	0.38(0.21–0.70)	0.25(0.11–0.44)	0.40(0.22–0.75)	0.43(0.31–1.29)	0.002
**Severe**					
	Total	Youth (<30 yr)	Middle age (30–60 yr)	Senior (> 60 yr)	P value
	(n = 53)	(n = 2)	(n = 29)	(n = 22)	
**Symptoms and signs**					
Diarrhea	1(1.89%)	1(50.00%)	0	0	0
Chills	2(3.77%)	1(50.00%)	1(3.45%)	0	0.002
**Laboratory findings**					
Mean Hemoglobin Concentration, G/L	336.00(324.00–340.00)	343.00(322.00-)	337.00(331.00–340.00)	328.00(321.00–336.00)	0.032

**Fig 1:**
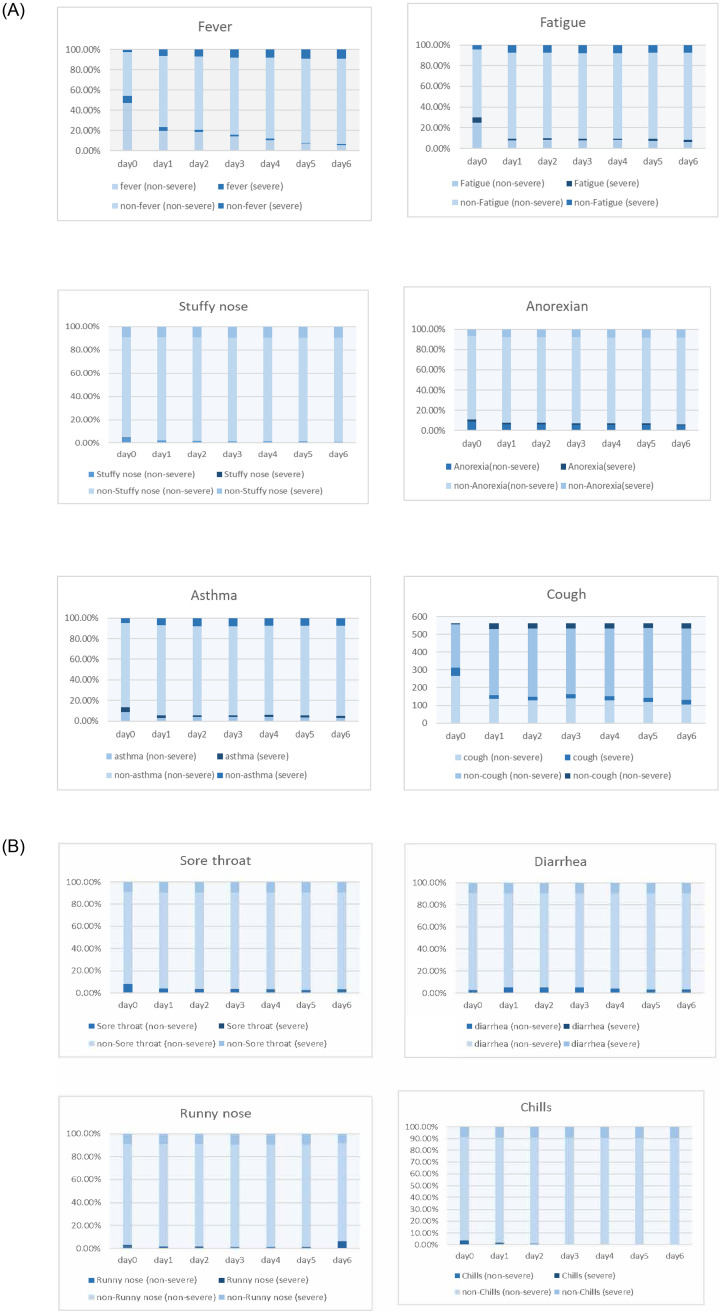
Clinical courses of different symptoms in severe and non-severe patients.

For the risk factor analysis, age, symptoms (fever, fatigue, asthma, cough, anorexia), physical and chemical indexes (cystatin C, HDL, count of WBC, neutrophil count, creatinine, total bilirubin, lymphocyte percentage, monocyte count, albumin, total bile acid, hematocrit (35–45), neutrophil percentage>75, prealbumin<150, Lymphocyte percentage<20, total bile acid<20, Lymphocyte count<1.1 and glucose) were associated with severe cases in univariable analysis (Table 4). Based on the odds ratio of above factors, we used multivariable logistic regression methods for further analysis, and found age, count of WBC, glucose, fever and asthma were associated with the severity of disease (Table 4). When adjusting for study center, the generalized linear model also showed similar results (S4 Table). The clinical dynamic profiles of main symptoms of non-severe and severe patients were shown in Fig 1.

**Table 4:** Risk factors associated with disease severity.

	Univariable OR (95% CI)	p value	Multivariable OR (95% CI)	p value
**Demographics**				
Age	1.06(1.04, 1.07)	< .0001	1.12(1.00, 1.26)	0.0441
Gender	1.22(0.69, 2.14)	0.4981		
**Symptoms and signs**				
Fever	3.23(1.78, 5.87)	0.0001	27.46(1.21, 622.50)	0.0375
Fatigue	2.94(1.61, 5.36)	0.0004		
Asthma	7.14(3.82, 13.34)	< .0001	28.49(1.81, 448.81)	0.0172
Cough	4.11(2.07, 8.17)	< .0001		
Anorexia	2.07(1.05, 4.07)	0.0344		
**Laboratory findings**				
Cystatin C	4.28(1.27, 14.37)	0.0186		
High density lipoprotein	2.31(0.99, 5.36)	0.0507		
White blood cell count	1.08(1.00, 1.17)	0.0427	1.64(1.16, 2.32)	0.0051
Total bilirubin	1.01(1.00, 1.03)	0.0541		
Creatinine	1.01(1.00, 1.02)	0.0236		
Neutrophil percentage	..	..		
<40	0.87(0.74, 1.03)	0.1135		
40–75	1.00(0.95, 1.05)	0.9585		
>75	1.13(1.03, 1.23)	0.0071		
Lymphocyte count	..	..		
<1.1	0.11(0.02, 0.88)	0.0377		
1.1–3.2	0.98(0.41, 2.31)	0.9584		
>3.2	1.14(0.81, 1.59)	0.4507		
Lymphocyte percentage	..	..		
<20	0.87(0.80, 0.95)	0.0016		
20–50	0.99(0.93, 1.05)	0.7552		
>50	..	..		
Monocyte percentage	0.87(0.78, 0.98)	0.0314		
Albumin	0.94(0.89, 0.99)	0.0309		
Total bile acid	..	..		
< = 20	0.81(0.68, 0.95)	0.0140		
>20	..	..		
Glucose	7.26(1.70, 30.95)	0.0073	14.89 (1.29, 171.13)	0.0301

OR = odds ratio.

## Discussion

Our retrospective study unraveled the clinic features and risk factors for laboratory-confirmed COVID-19 patients with different severity in China. We found that age, laboratory indicators (albumin, cystatin, white blood cell count, neutrophil count, lymphocyte percentage, mean hemoglobin, oxygen saturation and glucose), and major symptoms (fever, fatigue, cough, anorexia, diarrhea and asthma) were markedly correlated with the disease severity. Further analysis from the perspective of gender to detect the clinical symptoms, liver and kidney function, inflammatory and immune system indicators imply that more intensive health surveillance and preventions should be focused on male cases in severe group to avoid the deterioration of liver and kidney function and the aggravation of infection more efficiently. Meanwhile, female patients with persistent cough in severe group indicate the progress of illness.

In view of the importance of the above factors, advanced age, count of WBC, glucose, fever and asthma could be considered as risk factors for the severity of COVID-19 though multivariable logistic regression model, which showed great significance to prevent COVID-19 patients from turning to critical cases during treatment.

Current mainstream data suggested that patients with severe or critically ill diseases who require intensive care were usually older, with a median age of 60 [21]. Our study has also shown that median age of patients (median age = 59.00) with severe illness were significantly older than non-severe patients (median age = 46.00) [6, 22]. The age-dependent decreases in cellular and humoral immune function in elderly patients have been reported before, especially with regard to adaptive immune function [23]. We also found that the elderly had higher levels of low-density lipoprotein, red blood cell distribution width and D-dimer level than other age groups, suggesting that these patients might have coagulation dysfunction. Moreover, the worse condition of older COVID-19 patients could also be attributed to underlying comorbidities. While the severity of the disease increases from the age of 40 years, population above the age of 60 years and those with underlying comorbidity including cardiovascular diseases, diabetes and other diseases are at the highest risk [20]. In addition, patients with underlying cardiovascular disease were more likely to have serious consequences of COVID-19 [24]. A recent study also illustrated cardiovascular comorbidities can increase the risk of COVID-19 infection, which lead to poorer prognosis [25]. Due to morphologic and hemodynamic damage to heart tissues, cardiac insufficiency in COVID-19 patients with acute coronary syndrome may occur rapidly, leading to a sudden deterioration and fatal complications [26]. Taken together, the need for early monitoring and supportive care in immune function, coagulation dysfunction and underlying comorbidity should be addressed in these older patients during the hospitalization.

Inflammatory indicators such as count of WBC, neutrophil count and lymphocyte percentage were shown significant differences between the severe and non-severe groups. Additionally, we found that female patients had faster ESR and lower inflammatory markers (WBC and neutrophils). To our knowledge, when the virus invades the body, the immune system is activated [27]. Recently, Lymphocytopenia was observed in most severe COVID-19 patients [28]. It was also reported in patients with severe acute respiratory syndrome caused by SARS virus, with a prevalence rate of 69.6% [29, 30]. SARS infection can directly inhibit bone marrow or induce immune-mediated lymphocyte destruction, resulting in lymphocytopenia [31]. SARS-CoV-2 may have an inherent mechanism similar to that of SARS virus, including direct infection, lymphocyte destruction [32], and cytokine-mediated lymphocyte destruction [33, 34]. The initial decrease of circulating lymphocytes at the beginning of SARS-CoV-2 infection may be related to a separate process. Firstly, lymphocytes were recruited from the peripheral circulation to infected and inflammatory areas. Autopsy reports of patients with COVID-19 showed interstitial inflammatory exudation dominated by lymphocytes in both lungs [35]. Secondly, SRAS-CoV-2 might induce some stimuli similar to SARS and MERS, triggering lymphocyte apoptosis [31, 36]. As for the gender difference of patients, although the mechanisms is not clear, however, it may help to provide a reference for doctors to make better decision in the process of clinical management. Specific treatments should be selected according to gender and age differences in advance, and possible targeted measures should be taken early according to changes in laboratory indicators (especially inflammatory indicators) of COVID-19 patients.

In consistence with previous studies [37], our study indicated that glucose was an important factor for the severity of COVID-19, which might also play a critical role in the pathogenesis of infectious diseases. Similar with a study of SARS, the researchers found that even non-severe patients had higher fasting blood glucose levels if they were not treated with glucocorticoids [38]. The virus infection could cause the sharp fluctuation of the blood glucose level of the diabetic patients, which affected the recovery of the patients. Therefore, diabetes mellitus complicated with SARS-CoV-2 pneumonia may form a vicious circle, which is not conducive to the prognosis of COVID-19 [39]. Previous studies have reported some viruses can directly cause pancreatic β-cell damage [40] and the expression of ACE2 as a SARS-CoV-2 receptor in pancreatic endocrine tissue is higher than that in exocrine tissue [41]. Previous studies also reported that two individuals developed transient acute insulin-dependent diabetes mellitus after being infected with chickenpox [42]. Thus, considering that SARS-CoV-2 binds to ACE2 and enters infected cells [43, 44], reducing the ACE2 expression [45], overactivation of the renin-angiotensin system may also increase the risk of adverse events in patients with COVID-19 and diabetes. Accordingly, the clinical use of renin-angiotensin system inhibitors may have a therapeutic effect on patients with COVID-19 and previous diabetes.

Other risk factors worth paying attention were specific clinical symptoms and signs. Fever and asthma was found the most representative symptoms of severity for COVID-19 in our study. When the patient’s immune response is low, it may manifest as normal body temperature. Shortness of breath or dyspnea suggests poor lung function and lacking of oxygen. In fact, alveolar membrane rupture and leakage caused by direct or indirect lung attacks caused by COVID-19 were the underlying mechanism of disease progression. Osmotic pulmonary edema begins with a mild cough, then rapidly develops into obvious dyspnea, causing mild cough, and then rapidly develops into obvious dyspnea, requiring early intubation [46]. Given that the high proportion of fever and asthma in severe patients, it could help clinicians identify which patients are at high risk, anticipate disease progression and take precautions during treatment.

The results of our study should be interpreted within the constraints of its limitations. Firstly, due to the retrospective study design, not all significant laboratory indicators were tested in such an emergency, which might play important role in predicting clinical progression. Secondly, there was inevitable possibility of information bias in these retrospective studies. Thirdly, dynamic monitoring is more meaningful for disease assessment and prediction. Here, we cannot fully describe the dynamic changes of some critical indicators of COVID-19 because of missing value in such urgent epidemic situation.

Collectively, the current study provides evidences that advanced age, count of WBC, glucose, fever and asthma were the most common risk factors affecting the severity of COVID-19 infection. Moreover, focusing on different gender and age groups of COVID-19 patients, some specific clinical features should be paid more attention, which would be of great significance in control and targeted intervention of COVID-19 disease worldwide.

## Supporting information

S1 Table Distribution of source hospitals for 562 COVID-19 patients. (DOCX)

S2 Table Comorbidities of enrolled patients. (DOCX)

S3 Table Demographic, clinical, laboratory, and radiographic findings of patients on admission. (DOCX)

S4 Table Risk factors associated with disease severity for study patients in generalized linear model. (DOCX)

S1 Dataset (XLSX)
