# Supplementary material for: Clinical features and risk factors for severe inpatients with COVID-19: A retrospective study in China
Source: PLoS One. 2020 Dec 17;15(12):e0244125. doi: 10.1371/journal.pone.0244125 (PMC7745975; doi:10.1371/journal.pone.0244125)
Supplement: S1 Table — (DOCX) [file pone.0244125.s001.docx]

**S1 Table. Distribution of source hospitals for 562 COVID-19 patients**

| **Hospital** | **Case** | **Percentage** |
| --- | --- | --- |
| Suining Central Hospital in Sichuan Province Suining city | 10 | 7.80% |
| Kang'an Hospital in Mudanjiang City Heilongjiang Province | 18 | 3.20% |
| The 2th Hospital in Daqing City Heilongjiang | 19 | 3.38% |
| Chongqing Traditional Chinese Medicine Hospital | 24 | 4.27% |
| The First Hospital of Qiqihar in Heilongjiang Province | 28 | 4.98% |
| The Infectious Disease Hospital of Jinzhong City in Shanxi Province | 32 | 5.69% |
| The people's hospital of guangzi zhuang autonomous region | 37 | 6.58% |
| No.1 Hospital In Suihua City in Heilongjiang Province | 45 | 8.01% |
| Anhui Traditional Chinese Medicine Hospital in Heifei city Anhui Province | 47 | 8.36% |
| The Affiliated Hospital(group) of Putian University in Putian city Fujian Province | 51 | 9.07% |
| The Infectious Hospital of Harbin in Heilognjiang Province | 68 | 12.10% |
| The Hospital Affiliated of Southwest Medical University in Luzhou city Sichuan Province | 183 | 32.56% |
| Total | 562 | 100% |
