# Supplementary material for: Clinical features and risk factors for severe inpatients with COVID-19: A retrospective study in China
Source: PLoS One. 2020 Dec 17;15(12):e0244125. doi: 10.1371/journal.pone.0244125 (PMC7745975; doi:10.1371/journal.pone.0244125)
Supplement: S2 Table — (DOCX) [file pone.0244125.s002.docx]

**S2 Table. Comorbidities of enrolled patients**

| **Variable** | **Count** |
| --- | --- |
| Hypertension | 84 |
| Diabetes | 48 |
| Coronary heart disease | 15 |
| Cerebral vascular disease | 9 |
| Lung diseases | 6 |
| Other | 66 |
| Hypertension + Diabetes | 15 |
| Hypertension + CHD | 7 |
| Hypertension + Cerebral vascular disease | 4 |
| Diabetes + CHD | 4 |
| Hypertension + Diabetes + CHD | 4 |
| Hypertension + Diabetes + CHD + Other | 2 |
| Abbreviation: CHD, coronary heart disease. | |
