# Supplementary material for: Clinical features and risk factors for severe inpatients with COVID-19: A retrospective study in China
Source: PLoS One. 2020 Dec 17;15(12):e0244125. doi: 10.1371/journal.pone.0244125 (PMC7745975; doi:10.1371/journal.pone.0244125)
Supplement: S3 Table — (DOCX) [file pone.0244125.s003.docx]

**S3 Table. Demographic, clinical, laboratory, and radiographic findings of patients on admission**

|  | **Total** | **Non-severe** | **Severe** | **p value** |
| --- | --- | --- | --- | --- |
|  | **(n=562)** | **(n=509)** | **(n=53)** |  |
| **Demographics** | | | | |
| Age, years | 47.00((35.00-57.00) | 46.00(33.00-56.00) | 59.00(48.00-65.00) | <0.0001 |
| Sex | .. | .. | .. |  |
| Female | 272(48.40%) | 244(47.94%) | 28(52.83%) | 0.498 |
| Male | 290(51.60%) | 265(52.06%) | 25(47.17%) |  |
| Epidemic area exposure history | 171(30.42%) | 164(32.22%) | 7(13.20%) | 0.004 |
| History of close contact with patients | 378(67.26%) | 346(67.98%) | 32(60.38%) | 0.350 |
| **Symptoms and signs** | | | | |
| Fever | 226(40.21%) | 191(37.52%) | 35(66.04%) | <0.0001 |
| Fatigue | 107(19.04%) | 87(17.09%) | 20(37.74%) | <0.0001 |
| Cough | 287(51.07%) | 245(48.13%) | 42(79.25%) | <0.0001 |
| Anorexia | 82(14.59%) | 69(13.56%) | 13(24.53%) | 0.031 |
| Diarrhea | 59(10.50%) | 58(11.39%) | 1(1.89%) | 0.032 |
| Asthma | 68(12.10%) | 46(9.04%) | 22(41.51%) | <0.0001 |
| Dry cough | 180(32.03%) | 157(30.84%) | 23(43.40%) | 0.062 |
| Stuffiness | 21(3.74%) | 20(3.93%) | 1(1.89%) | 0.456 |
| Runny nose | 18(3.20%) | 16(3.14%) | 2(3.77%) | 0.804 |
| Sore throat | 35(6.23%) | 34(6.68%) | 1(1.89%) | 0.169 |
| Chills | 19(3.38%) | 17(3.34%) | 2(3.77%) | 0.868 |
| **Comorbidities** | | | | |
| Hypertension | 84(14.95%) | 78(15.32%) | 6(11.32%) | 0.437 |
| Diabetes | 48(8.54%) | 42(8.25%) | 6(11.32%) | 0.447 |
| Coronary heart disease | 15(2.67%) | 14(2.75%) | 1(1.89%) | 0.710 |
| Cerebral vascular disease | 9(1.60%) | 9(1.77%) | 0 | 0.329 |
| Lung diseases | 6(1.07%) | 6(1.18%) | 0 | 0.427 |
| Others (thyroid disease, enteritis, fracture, etc.) | 66(11.74%) | 59(11.59%) | 7(13.21%) | 0.728 |
| **Laboratory findings** | | | | |
| Alanine aminotransferase,IU/L | 24.00(16.00-39.00) | 24.00(16.00-40.00) | 24.00(16.75-35.00) | 0.893 |
| ＜7 | 6(1.07%) | 6(1.18%) | 0(0.00%) | 0.503 |
| 7-40 | 331(58.90%) | 297(58.35%) | 34(64.15%) |  |
| ＞40 | 107(19.04%) | 99(19.45%) | 8(15.09%) |  |
| Aspartate aminotransferase,IU/L | 21.90(17.00-31.00) | 21.80(17.00-32.00) | 22.00(17.00-29.25) | 0.780 |
| ＜12 | 16(2.85%) | 14(2.75%) | 2(3.77%) | 0.577 |
| 12-35 | 362(64.41%) | 326(64.05%) | 36(67.92%) |  |
| ＞35 | 65(11.57%) | 61(11.98%) | 4(7.55%) |  |
| γ-glutamyl transpeptidase,IU/L | 30.00(19.00-57.50) | 30.27(19.00-57.09) | 28.00(22.00-64.00) | 0.608 |
| ＜7 | 1(0.18%) | 1(0.20%) | 0(0.00%) | 0.944 |
| 7-45 | 289(51.42%) | 261(51.28%) | 28(52.83%) |  |
| ＞45 | 151(26.87%) | 136(26.72%) | 15(28.30%) |  |
| Alkaline phosphatase,IU/L | 66.45(51.00-86.00) | 67.30(51.00-86.80) | 62.00(50.00-76.90) | 0.158 |
| ＜50 | 98(17.44%) | 88(17.29%) | 10(18.87%) | 0.304 |
| 50-75 | 174(30.96%) | 153(30.06%) | 21(39.62%) |  |
| ＞75 | 168(29.89%) | 156(30.65%) | 12(22.64%) |  |
| Cholinesterase,IU/L | 7445.00(6205.00-8540.00) | 7488.50(6289.50-8544.50) | 7083.00(5089.00-7958.00) | 0.161 |
| ＜4000 | 8(1.42%) | 6(1.18%) | 2(3.77%) | 0.521 |
| 4000-12000 | 206(36.65%) | 181(35.56%) | 25(47.17%) |  |
| ＞12000 | 1(0.18%) | 1(0.20%) | 0 |  |
| Lactate dehydrogenase,IU/L | 193.00(155.12-233.75) | 193.55(155.00-233.25) | 186.00(160.75-245.00) | 0.749 |
| 20-240 | 303(53.91%) | 276(54.22%) | 27(50.94%) | 0.730 |
| ＞240 | 89(15.84%) | 80(15.72%) | 9(16.98%) |  |
| α-hydroxybutyrate dehydrogenase,IU/L | 157.77(133.75-200.65) | 157.77(134.14-200.09) | 158.00(126.75-219.44) | 0.989 |
| ＜72 | 3(0.53%) | 3(0.59%) | 0 | 0.589 |
| 72-182 | 108(19.22%) | 95(18.66%) | 13(24.53%) |  |
| ＞182 | 55(9.79%) | 46(9.04%) | 9(16.98%) |  |
| ApoA-1,g/L | 0.95(0.75-1.11) | 0.95(0.74-1.12) | 0.91(0.80-1.10) | 0.959 |
| ＜1 | 68(12.10%) | 59(11.59%) | 9(16.98%) | 0.606 |
| 1-1.6 | 49(8.72%) | 45(8.84%) | 4(7.55%) |  |
| ＞1.6 | 2(0.36%) | 2(0.39%) | 0 |  |
| Apo-B,g/L | 0.82(0.61-1.00) | 0.82(0.60-0.99) | 0.91(0.69-1.23) | 0.152 |
| ＜0.6 | 26(4.63%) | 26(5.11%) | 0 | 0.118 |
| 0.6-1.1 | 74(13.17%) | 64(12.57%) | 10(18.87%) |  |
| B＞1.1 | 18(3.20%) | 15(2.95%) | 3(5.66%) |  |
| Total protein,g/L | 67.77(62.00-73.90) | 67.80(62.00-73.91) | 66.50(61.76-73.43) | 0.559 |
| ＜65 | 185(32.92%) | 166(32.61%) | 19(35.85%) | 0.307 |
| 65-83 | 289(51.42%) | 260(51.08%) | 29(54.72%) |  |
| ＞83 | 21(3.74%) | 21(4.13%) | 0 |  |
| Albumin,g/L | 39.70(35.88-43.93) | 39.95(36.00-44.00) | 37.97(33.93-42.56) | 0.037 |
| ＜40 | 256(45.55%) | 225(44.20%) | 31(58.49%) | 0.137 |
| 40-55 | 231(41.10%) | 215(42.24%) | 16(30.19%) |  |
| ＞55 | 2(0.36%) | 2(0.39%) | 0(0.00%) |  |
| Globulin,g/L | 28.00(25.00-31.00) | 28.00(25.00-31.00) | 27.98(24.00-30.86) | 0.748 |
| ＜20 | 22(3.91%) | 21(4.13%) | 1(1.89%) | 0.562 |
| 20-40 | 462(82.21%) | 417(81.93%) | 45(84.91%) |  |
| ＞40 | 13(2.31%) | 11(2.16%) | 2(3.77%) |  |
| Prealbumin,g/L | 196.80(129.50-252.00) | 196.00(132.50-253.00) | 197.00(113.85-250.60) | 0.726 |
| ＜150 | 83(14.77%) | 70(13.75%) | 13(24.53%) | 0.208 |
| 150-400 | 194(34.52%) | 174(34.18%) | 20(37.74%) |  |
| Total bilirubin,umol/L | 11.50(7.15-16.67) | 11.50(7.18-16.30) | 12.50(6.95-21.12) | 0.357 |
| ＜1.7 | 1(0.18%) | 1(0.20%) | 0 | 0.157 |
| 1.7-20 | 412(73.31%) | 378(74.26%) | 34(64.15%) |  |
| ＞20 | 80(14.23%) | 68(13.36%) | 12(22.64%) |  |
| Direct bilirubin,umol/L | 3.49(2.16-5.44) | 3.49(2.12-5.40) | 3.61(2.70-6.03) | 0.240 |
| ＜1 | 25(4.45%) | 25(4.91%) | 0 | 0.157 |
| 1-14 | 440(78.29%) | 394(77.41%) | 46(86.79%) |  |
| ＞14 | 7(1.25%) | 7(1.38%) | 0(0.00%) |  |
| Indirect bilirubin,umol/L | 7.50(4.75-11.40) | 7.50(4.80-11.23) | 8.02(4.00-13.33) | 0.719 |
| ＜15 | 413(73.49%) | 376(73.87%) | 37(69.81%) | 0.062 |
| ＞15 | 60(10.68%) | 50(9.82%) | 10(18.87%) |  |
| Low-density lipoprotein,mmol/L | 2.42(1.96-2.99) | 2.43(1.96-2.96) | 2.27(1.96-3.24) | 0.998 |
| ＜4.11 | 250(44.48%) | 226(44.40%) | 24(45.28%) | 0.515 |
| ＞4.11 | 4(0.71%) | 4(0.79%) | 0 |  |
| High density lipoprotein,mmol/L | 1.03(0.85-1.25) | 1.01(0.84-1.25) | 1.06(0.91-1.28) | 0.253 |
| ＜0.9 | 78(13.88%) | 72(14.15%) | 6(11.32%) | 0.129 |
| 0.9-2.5 | 177(31.49%) | 159(31.24%) | 18(33.96%) |  |
| ＞2.5 | 2(0.36%) | 1(0.20%) | 1(1.89%) |  |
| Total cholesterol,mmol/L | 4.11(3.55-4.82) | 4.10(3.55-4.80) | 4.34(3.47-4.86) | 0.741 |
| ＜2.8 | 21(3.74%) | 20(3.93%) | 1(1.89%) | 0.588 |
| 2.8-6 | 310(55.16%) | 278(54.62%) | 32(60.38%) |  |
| ＞6 | 21(3.74%) | 18(3.54%) | 3(5.66%) |  |
| Creatine kinase,IU/L | 56.00(37.00-84.60) | 56.00(37.00-84.00) | 51.00(37.06-94.76) | 0.959 |
| ＜25 | 33(5.87%) | 30(5.89%) | 3(5.66%) | 0.726 |
| 25-190 | 319(56.76%) | 287(56.39%) | 32(60.38%) |  |
| ＞190 | 21(3.74%) | 20(3.93%) | 1(1.89%) |  |
| Amylase,U/L | 54.00(42.31-67.45) | 53.00(42.37-67.00) | 58.00(38.00-78.83) | 0.536 |
| ＜140 | 175(31.14%) | 151(29.67%) | 24(45.28%) |  |
| Lipase,U/L | 39.70(29.65-50.40) | 39.15(30.48-50.60) | 41.70(21.70-51.25) | 0.595 |
| ＜60 | 59(10.50%) | 51(10.02%) | 8(15.09%) | 0.834 |
| ＞60 | 6(1.07%) | 5(0.98%) | 1(1.89%) |  |
| Adenosine deaminase,IU/L | 11.70(9.00-14.00) | 11.79(9.00-14.00) | 11.62(9.50-14.10) | 0.851 |
| ＜4 | 1(0.18%) | 1(0.20%) | 0 | 0.635 |
| 4-20 | 159(28.29%) | 138(27.11%) | 21(39.62%) |  |
| ＞20 | 5(0.89%) | 5(0.98%) | 0 |  |
| Triglyceride,mmol/L | 1.43(0.96-2.05) | 1.44(0.97-2.08) | 1.31(0.78-2.00) | 0.427 |
| 0.24-1.86 | 225(40.04%) | 201(39.49%) | 24(45.28%) | 0.590 |
| ＞1.86 | 103(18.33%) | 94(18.47%) | 9(16.98%) |  |
| Triacylglycerol,mmol/L | 1.43(0.88-2.67) | 1.43(0.80-2.10) | 1.69(0.94-) | 0.634 |
| ＜1.7 | 9(1.60%) | 8(1.57%) | 1(1.89%) | 0.849 |
| ＞1.7 | 7(1.25%) | 6(1.18%) | 1(1.89%) |  |
| Urea,mmol/L | 3.90(3.10-4.97) | 3.90(3.08-4.84) | 4.32(3.19-5.51) | 0.171 |
| ＜2.5 | 42(7.47%) | 37(7.27%) | 5(9.43%) | 0.271 |
| 2.5-8.2 | 376(66.90%) | 342(67.19%) | 34(64.15%) |  |
| ＞8.2 | 14(2.49%) | 11(2.16%) | 3(5.66%) |  |
| Uric acid,umol/L | 278.47(214.00-348.36) | 278.97(218.00-348.10) | 265.47(170.00-354.00) | 0.479 |
| ＜140 | 29(5.16%) | 24(4.72%) | 5(9.43%) | 0.140 |
| 140-440 | 403(71.71%) | 368(72.30%) | 35(66.04%) |  |
| ＞440 | 38(6.76%) | 32(6.29%) | 6(11.32%) |  |
| Creatinine,umol/L | 62.00(52.00-75.18) | 61.66(51.63-75.00) | 67.20(54.85-78.25) | 0.119 |
| ＜53 | 127(22.60%) | 118(23.18%) | 9(16.98%) | 0.340 |
| 53-97 | 327(58.19%) | 293(57.56%) | 34(64.15%) |  |
| ＞97 | 18(3.20%) | 15(2.95%) | 3(5.66%) |  |
| Complement C1q,mg/L | 157.20(143.16-166.53) | 157.20(144.21-167.26) | 150.16(134.51-) | 0.427 |
| ＜159 | 8(1.42%) | 7(1.38%) | 1(1.89%) | 1.000 |
| 159-233 | 8(1.42%) | 7(1.38%) | 1(1.89%) |  |
| α-L-fucosidase,U/L | 26.00(21.37-29.98) | 26.00(21.30-29.90) | 26.20(21.50-30.50) | 0.955 |
| ＜40 | 107(19.04%) | 95(18.66%) | 12(22.64%) | 0.992 |
| ＞40 | 9(1.60%) | 8(1.57%) | 1(1.89%) |  |
| 5‘nuclease,U/L | 3.00(2.38-5.73) | 3.00(2.13-5.38) | 12.00（3.00-） | 0.331 |
| ＜10 | 29(5.16%) | 28(5.50%) | 1(1.89%) | 0.000 |
| ＞10 | 1(0.18%) | 0(0.00%) | 1(1.89%) |  |
| Cystatin C,mg/L | 0.94(0.80-1.12) | 0.94(0.80-1.10) | 1.00(0.81-1.36) | 0.187 |
| ＜0.54 | 2(0.36%) | 2(0.39%) | 0 | 0.017 |
| 0.54-1.5 | 182(32.38%) | 165(32.42%) | 17(32.08%) |  |
| ＞1.5 | 11(1.96%) | 7(1.38%) | 4(7.55%) |  |
| Urea/Creatinine | 61.63(5.55-90.47) | 60.83(3.78-89.44) | 76.15(45.91-99.70) |  |
| Glomerular filtration rate | 111.25(102.37-120.79) | 110.82(102.37-122.09) | 117.01(100.41-117.80) | 0.793 |
| ＞90 | 63(11.21%) | 55(10.81%) | 8(15.09%) | 0.353 |
| ＜90 | 6(1.07%) | 6(1.18%) | 0 |  |
| Glucose,mmol/L | 5.57(4.89-6.87) | 5.57(4.88-6.79) | 5.68(4.92-7.64) | 0.400 |
| ＜3.57 | 4(0.71%) | 4(0.79%) | 0 | 0.599 |
| 3.57-6.16 | 250(44.48%) | 227(44.60%) | 23(43.40%) |  |
| ＞6.16 | 147(26.16%) | 130(25.54%) | 17(32.08%) |  |
| K,mmol/L | 3.90(3.58-4.20) | 3.90(3.58-4.21) | 3.88(3.60-4.14) | 0.483 |
| ＜3.5 | 78(13.88%) | 72(14.15%) | 6(11.32%) | 0.598 |
| 3.5-5.3 | 382(67.97%) | 345(67.78%) | 37(69.81%) |  |
| ＞5.3 | 7(1.25%) | 7(1.38%) | 0 |  |
| Na,mmol/L | 139.00(137.00-140.9.00) | 139.00(137.00-140.98) | 137.75(136.00-140.11) | 0.064 |
| ＜137 | 103(18.33%) | 89(17.49%) | 14(26.42%) | 0.262 |
| 137-147 | 351(62.46%) | 319(62.67%) | 32(60.38%) |  |
| ＞147 | 8(1.42%) | 8(1.57%) | 0(0.00%) |  |
| Ca,mmol/L |  | | | |
| ＜2.1 | 90(16.01%) | 77(15.13%) | 13(24.3%) | 0.161 |
| 2.1-2.8 | 341(60.68%) | 312(61.30%) | 29(54.72%) |  |
| ＞2.8 | 7(1.25%) | 7(1.38%) | 0 |  |
| Cl,mmol/L | 102.80(100.00-104.90) | 102.90(100.00-104.90) | 102.00(98.00-104.55) | 0.104 |
| C02,mmol/L | 25.19(24.00-27.00) | 25.10(24.00-27.00) | 26.00(23.42-27.98) | 0.285 |
| ＜18 | 4(0.71%) | 4(0.79%) | 0(0.00%) | 0.578 |
| 18-31 | 282(50.18%) | 255(50.10%) | 27(50.94%) |  |
| ＞31 | 12(2.14%) | 10(1.96%) | 2(3.77%) |  |
| P,mmol/L | 1.03(0.87-1.22) | 1.04(0.87-1.21) | 1.01(0.85-1.30) | 0.855 |
| ＜0.73 | 18(3.20%) | 16(3.14%) | 2(3.77%) | 0.627 |
| 0.73-1.55 | 191(33.99%) | 178(34.97%) | 13(24.53%) |  |
| ＞1.55 | 6(1.07%) | 6(1.18%) | 0 |  |
| Total osmotic pressure,mOSM/l | 295.10(291.00-299.00) | 295.10(291.00-298.55) | 297.50(290.50-302.35) | 0.389 |
| ＜280 | 4(0.71%) | 9(1.77%) | 0 | 0.567 |
| 280-320 | 79(14.06%) | 73(14.34%) | 6(11.32%) |  |
| Blood lactic acid,mmol/L | 2.90(2.90-2.90) | 2.90(2.90-2.90) | .. |  |
| Anion gap,mmol/L | 10.50(8.85-12.20) | 10.40(8.73-12.20) | 11.35(9.00-12.75) | 0.589 |
| ＜10 | 45(8.01%) | 42(8.25%) | 3(5.66%) | 0.685 |
| 10-14 | 57(10.14%) | 52(10.22%) | 5(9.43%) |  |
| ＞14 | 7(1.25%) | 7(1.38%) | 0 |  |
| White blood cell count, 10^9/L | 5.86(4.49-7.56) | 5.80(4.46-7.47) | 6.65(4.61-9.30) | 0.043 |
| ＜3.5 | 45(8.01%) | 43(8.45%) | 2(3.77%) | 0.015 |
| 3.5-9.5 | 402(71.53%) | 368(72.30%) | 34(64.15%) |  |
| ＞9.5 | 50(8.90%) | 40(7.86%) | 10(18.87%) |  |
| Neutrophil count, 10^9/L | 3.70(2.74-5.32) | 3.62(2.70-5.20) | 4.86(3.17-7.69) | 0.013 |
| ＜1.8 | 36(6.41%) | 34(6.68%) | 2(3.77%) | 0.013 |
| 1.8-6.3 | 381(67.79%) | 352(69.16%) | 29(54.72%) |  |
| ＞6.3 | 79(14.06%) | 65(12.77%) | 14(26.42%) |  |
| Neutrophil percentage,% | 65.45(56.88-74.93) | 65.30(56.80-74.10) | 68.10(57.30-81.10) | 0.091 |
| ＜40 | 16(2.85%) | 14(2.75%) | 2(3.77%) | 0.131 |
| 40-75 | 359(63.88%) | 331(65.03%) | 28(52.83%) |  |
| ＞75 | 123(21.89%) | 106(20.83%) | 17(32.08%) |  |
| Lymphocyte count,10^9/L | 1.38(0.96-1.80) | 1.38(1.00-1.84) | 1.36(0.71-1.76) | 0.204 |
| ＜1.1 | 156(27.76%) | 137(26.92%) | 19(35.85%) | 0.359 |
| 1.1-3.2 | 331(58.90%) | 304(59.72%) | 27(50.94%) |  |
| ＞3.2 | 18(3.20%) | 16(3.14%) | 2(3.77%) |  |
| Lymphocyte percentage, % | 24.30(16.98-32.70) | 24.65(17.88-33.00) | 20.90(10.40-29.18) | 0.010 |
| ＜20 | 174(30.96%) | 151(29.67%) | 23(43.40%) | 0.083 |
| 20-50 | 322(57.30%) | 297(58.35%) | 25(47.17%) |  |
| ＞50 | 10(1.78%) | 10(1.96%) | 0 |  |
| Monocyte count,10^9/L | 0.43(0.32-0.56) | 0.42(0.32-0.56) | 0.48(0.31-0.59) | 0.494 |
| ＜1.1 | 421(74.91%) | 381(74.85%) | 40(75.47%) | 0.441 |
| 1.1-3.2 | 5(0.89%) | 5(0.98%) | 0 |  |
| ＞3.2 | 4(0.71%) | 3(0.59%) | 1(1.89%) |  |
| Monocyte percentage | 7.40(5.90-9.30) | 7.50(5.98-9.40) | 6.80(4.70-8.90) | 0.061 |
| ＜3 | 427(75.98%) | 386(75.83%) | 41(77.36%) | 0.690 |
| 3-10 | 6(1.07%) | 6(1.18%) | 0(0.00%) |  |
| ＞10 | 1(0.18%) | 1(0.20%) | 0(0.00%) |  |
| Eosinophil count,10^9/L | 0.04(0.01-0.09) | 0.04(0.01-0.09) | 0.02(0-0.10) | 0.274 |
| ＜0.02 | 1(0.18%) | 1(0.20%) | 0 | 0.242 |
| 0.02-0.52 | 150(26.69%) | 130(25.54%) | 20(37.74%) |  |
| ＞0.52 | 275(48.93%) | 254(49.90%) | 21(39.62%) |  |
| Eosinophil percentage,% | 0.60(0.10-1.60) | 0.65(0.10-1.60) | 0.30(0-1.85) | 0.086 |
| ＜0.4 | 173(30.78%) | 151(29.67%) | 22(41.51%) | 0.157 |
| 0.4-8 | 254(45.20%) | 235(46.17%) | 19(35.85%) |  |
| ＞8 | 4(0.71%) | 4(0.79%) | 0 |  |
| Basophil count,10^9/L | 0.01(0.01-0.02) | 0.01(0.01-0.02) | 0.01(0.01-0.03) | 0.270 |
| Basophil percentage | 0.20(0.10-0.40) | 0.20(0.10-0.40) | 0.20(0.10-0.40) | 0.989 |
| ＜1 | 422(75.09%) | 381(74.85%) | 41(77.36%) | 0.464 |
| ＞1 | 5(0.89%) | 5(0.98%) | 0 |  |
| Red blood cell count,10^12/L | 4.46(4.07-4.85) | 4.47(4.06-4.86) | 4.42(4.22-4.84) | 0.919 |
| ＜3.8 | 65(11.57%) | 58(11.39%) | 7(13.21%) | 0.667 |
| 3.8-5.1 | 367(65.30%) | 332(65.23%) | 35(66.04%) |  |
| ＞5.1 | 75(13.35%) | 70(13.75%) | 5(9.43%) |  |
| Haemoglobin,g/L | 134.00(123.00-148.00) | 134.00(123.00-147.50) | 134.50(122.50-149.75) | 0.901 |
| ＜115 | 78(13.88%) | 71(13.95%) | 7(13.21%) | 0.897 |
| 115-150 | 315(56.05%) | 287(56.39%) | 28(52.83%) |  |
| ＞150 | 106(18.86%) | 95(18.66%) | 11(20.75%) |  |
| Hematocrit,% | 39.50(34.90-43.80) | 39.40(34.93-43.68) | 40.40(31.25-44.70) | 0.698 |
| ＜35 | 123(21.89%) | 111(21.81%) | 12(22.64%) | 0.946 |
| 35-45 | 273(48.58%) | 249(48.92%) | 24(45.28%) |  |
| ＞45 | 92(16.37%) | 84(16.50%) | 8(15.09%) |  |
| Average volume of red blood cells,fl | 90.20(87.00-93.40) | 90.05(86.90-93.40) | 91.50(88.10-93.05) | 0.227 |
| Mean hemoglobin,pg | 30.40(29.20-31.40) | 30.40(29.13-31.40) | 30.25(29.33-31.28) | 0.870 |
| ＜27 | 47(8.36%) | 45(8.84%) | 2(3.77%) | 0.051 |
| 27-34 | 441(78.47%） | 399(78.39%) | 42(79.25%) |  |
| ＞34 | 16(2.85%) | 12(2.36%) | 4(7.55%) |  |
| Mean hemoglobin concentration,g/L | 335.00(326.00-343.00) | 335.00(326.00-344.00) | 336.00(324.00-340.00) | 0.671 |
| ＜316 | 46(8.19%) | 43(8.45%) | 3(5.66%) | 0.746 |
| 316-354 | 435(77.40%) | 392(77.01%) | 43(81.13%) |  |
| ＞354 | 24(4.27%) | 22(4.32%) | 2(3.77%) |  |
| Red blood cell distribution width SD,fL | 41.00(38.30-44.00) | 40.95(38.10-44.00) | 41.00(39.20-43.30) | 0.470 |
| ＜39 | 133(23.67%) | 126(24.75%) | 7(13.21%) | 0.076 |
| 39-46 | 223(39.68%) | 196(38.51%) | 27(50.94%) |  |
| ＞46 | 71(12.63%) | 66(12.97%) | 5(9.43%) |  |
| Red blood cell distribution width CV,% | 12.50(12.00-13.28) | 12.50(12.00-13.30) | 12.60(12.00-13.20) | 0.810 |
| ＜11.5 | 27(4.80%) | 23(4.52%) | 4(7.55%) | 0.494 |
| 11.5-14.5 | 406(72.24% | 367(72.10%) | 39(3.58%) |  |
| ＞14.5 | 47(8.36%) | 44(8.64%) | 3(5.66%) |  |
| Platelet count,10^9/L | 202.00(154.00-260.00) | 201.00(152.00-258.50) | 214.00(158.00-276.25) | 0.412 |
| 125-350 | 470(83.63%) | 426(83.69%) | 44(83.02%) | 0.656 |
| ＞350 | 29(5.16%) | 27(5.30%) | 2(3.77%) |  |
| Platelet hematocrit,% | 0.21(0.17-0.26) | 0.21(0.17-0.26) | 0.24(0.17-0.31) | 0.087 |
| ＜0.17 | 9216.37%） | 86(16.90%) | 6(11.32%) | 0.070 |
| 0.17-0.39 | 276(49.11%) | 252(49.51%) | 24(45.28%) |  |
| ＞0.39 | 11(1.96%) | 8(1.57%) | 3(5.66%) |  |
| Mean platelet volume,fl | 10.10(9.30-11.10) | 10.10(9.30-11.10) | 9.90(9.35-11.05) | 0.836 |
| ＜9 | 79(14.06%) | 74(14.54%) | 5(9.43%) | 0.319 |
| 9-13 | 363(64.59%) | 327(64.24%) | 36(67.92%) |  |
| Platelet distribution width | 15.60(11.60-16.40) | 15.60(11.60-16.40) | 15.80(11.10-16.50) | 0.523 |
| <9 | 5(0.89%) | 5(0.98%) | 0 | 0.450 |
| 9~17 | 388(69.04%) | 356(69.94%) | 32(60.38%) |  |
| >17 | 56(9.96%) | 49(9.63%) | 7(13.21%) |  |
| Large platelet ratio,% | 27.20(22.35-34.15) | 27.40(22.15-34.65) | 25.30(22.60-32.40) | 0.521 |
| <13 | 3(0.53%) | 2(0.39%) | 1(1.89%) | 0.227 |
| 13~43 | 286(50.89%) | 261(51.28%) | 25(47.17%) |  |
| >43 | 36(6.41%) | 31(6.09%) | 5(9.43%) |  |
| Naive granulocyte percentage,% | 0(0.03-0.38) | 0.10(0.03-0.38) | 0.08(0.03-0.40) | 0.838 |
| ＜4 | 39(6.94%) | 35(6.88%) | 4(7.55%) | 0.736 |
| ＞4 | 1(0.18%) | 1(0.20%) | 0 |  |
| Activated partial thromboplastin time,s | 30.80(26.20-35.10) | 30.60(26.10-35.55) | 31.30(27.55-34.45) | 0.723 |
| ＜20 | 6(1.07%) | 5(0.98%) | 1(1.89%) | 0.347 |
| 20~40 | 232(41.28%) | 205(40.28%) | 27(50.94%) |  |
| ＞40 | 30(5.34%) | 29(5.70%) | 1(1.89%) |  |
| Fibrinogen,g/L | 3.20(2.51-3.97) | 3.20(2.51-3.97) | 3.31(2.37-4.37) | 0.872 |
| ＜1.7 | 9(1.60%) | 8(1.57%) | 1(1.89%) | 0.906 |
| 1.7~4 | 205(36.48%) | 185(36.35%) | 20(37.74%) |  |
| ＞4 | 69(12.28%) | 61(11.98%) | 8(15.09%) |  |
| Prothrombin time,s | 12.20(11.50-13.20) | 12.20(11.50-13.10) | 12.60(11.45-14.05) | 0.201 |
| ＜9.5 | 2(0.36%) | 2(0.39%) | 0 | 0.875 |
| 9.5~14.5 | 260(46.26%) | 233(45.78%) | 27(50.94%) |  |
| ＞14.5 | 22(3.91%) | 20(3.93%) | 2(3.77%) |  |
| D-dimer,ug/mL(ng/mL,mg/L) | 0.38(0.22-0.77) | 0.38(0.21-0.70) | 0.38(0.24-1.36) | 0.562 |
| ＜200 | 202(35.94%) | 179(35.17%) | 23(43.40%) | 0.720 |
| ＞200 | 1(0.18%) | 1(0.20%) | 0(0.00%) |  |
| Erythrocyte sedimentation rate,,MM/h | 23(10-38) | 24(10-38) | 11(5-28) | 0.227 |
| 0~20 | 59(10.50%) | 52(10.22%) | 7(13.21%) | 0.187 |
| ＞20 | 73(12.99%) | 69(13.56%) | 4(7.55%) |  |
| C-reactive protein,mg/L | 8.50(2.60-27.35) | 7.70(2.58-24.75) | 11.55(4.93-38.98) | 0.159 |
| ＜10 | 115(20.46%) | 106(20.83%) | 9(16.98%) | 0.159 |
| ＞10 | 94(16.73%) | 81(15.91%) | 13(24.53%) |  |
| Hypersensitive C-reactive protein,mg/L | 2.59(1.42-7.97) | 2.58(1.43-7.95) | 2.89(1.21-8.01) | 1.000 |
| ＜0.5 | 8(1.42%) | 8(1.57%) | 0 | 0.322 |
| ＞0.5 | 100(17.79%) | 89(17.49%) | 11(20.75%) |  |
| Procalcitonin,ng/ml | 0.09(0.05-0.14) | 0.09(0.05-0.14) | 0.09(0.04-0.14) | 0.911 |
| ＜0.5 | 114(20.28%) | 100(19.65%) | 14(26.42%) | 0.453 |
| ＞0.5 | 4(0.71%) | 3(0.59%) | 1(1.89%) |  |
| TroponinI | 0.06(0.05-4.02) | 2.04(0.05-5.62) | 0.01(0.01-0.01) | 0.130 |
| 0~0.1 | 4(0.71%) | 3(0.59%) | 1(1.89%) | 0.350 |
| ＞0.1 | 3(0.53%) | 3(0.59%) | 0(0.00%) |  |
| Cardiac troponin I,ug/L | 0.06(0.02-0.19) | 0.08(0.02-0.24) | 0.04(0.02-1.19) | 0.684 |
| 0~0.3 | 18(3.20%) | 13(2.55%) | 5(9.43%) | 0.910 |
| ＞0.3 | 4(0.71%) | 3(0.59%) | 1(1.89%) |  |
| Hypersensitive troponin, pg/ml | 0.90(0.03-9.60) | 2.62(0.03-9.83) | 0.90(0.90-0.90) | 1.000 |
| 0~14 | 21(3.74%) | 20(3.93%) | 1(1.89%) | 0.752 |
| ＞14 | 2(0.36%) | 2(0.39%) | 0(0.00%) |  |
| Myoglobin,ng/ml | 26.45(18.13-49.25) | 25.00(17.43-38.38) | 34.25(21.60-89.80) | 0.154 |
| ＜25 | 37(6.58%) | 34(6.68%) | 3(5.66%) | 0.472 |
| 25~38 | 22(3.91%) | 19(3.73%) | 3(5.66%) |  |
| ＞38 | 21(3.74%) | 17(3.34%) | 4(7.55%) |  |
| PH | 7.41(7.38-7.44) | 7.41(7.38-7.44) | 7.39(7.36-7.43) | 0.303 |
| ＜7.35 | 1(0.18%) | 1(0.20%) | 0 | 0.968 |
| 7.35~7.45 | 67(11.92%) | 63(12.38%) | 4(7.55%) |  |
| ＞7.45 | 16(2.85%) | 15(2.95%) | 1(1.89%) |  |
| Oxygen partial pressure | 91.70(74.00-116.50) | 91.90(74.10-122.50) | 89.90(51.35-100.50) | 0.307 |
| ＜80 | 33(5.87%) | 31(6.09%) | 2(3.77%) | 0.640 |
| 80~100 | 23(4.09%) | 21(4.13%) | 2(3.77%) |  |
| ＞100 | 34(6.05%) | 33(6.48%) | 1(1.89%) |  |
| Oxygen partial pressure (T correction),mmHg | 109.80(85.00-163.00) | 114.00(86.40-163.50) | 73.15(43.30-) | 0.185 |
| <75 | 6(1.07%) | 5(0.98%) | 1(1.89%) | 0.287 |
| 75~100 | 10(1.78%) | 10(1.96%) | 0(0.00%) |  |
| ＞100 | 27(4.80%) | 26(5.11%) | 1(1.89%) |  |
| Actual bicarbonate,mmol/L | 24.90(23.30-26.10) | 24.90(23.30-26.10) | 24.30(23.55-25.75) | 0.656 |
| ＜22 | 11(1.96%) | 11(2.16%) | 0 | 0.397 |
| 22~27 | 66(11.74%) | 61(11.98%) | 5(9.43%) |  |
| ＞27 | 12(2.14%) | 12(2.36%) | 0 |  |
| Standard bicarbonate,mmol/L | 24.50(23.45-26.78) | 24.50(23.50-25.85) | 23.70(22.60-) | 0.337 |
| ＜22 | 3(0.53%) | 3(0.59%) | 0 | 0.768 |
| 22~27 | 65(11.57%) | 62(12.18%) | 3(5.66%) |  |
| ＞27 | 8(1.42%) | 8(1.57%) | 0 |  |
| Whole blood remaining alkali,mmol/L | 0.20(-1.00-1.65) | 0.20(-1.00-1.78) | .. | 0.568 |
| ＜-3 | 3(0.53%) | 3(0.59%) | 0(0.00%) | 0.688 |
| `-3~3 | 78(13.88%) | 73(14.34%) | 5(9.43%) |  |
| ＞3 | 8(1.42%) | 8(1.57%) | 0(0.00%) |  |
| Arterial oxygen content | 16.80(14.65-19.20) | 16.80(14.70-19.20) | 16.8 | 0.934 |
| ＜6.7 | 1(0.18%) | 1(0.20%) | 0(0.00%) | 0.952 |
| 6.7~10.3 | 1(0.18%) | 1(0.20%) | 0(0.00%) |  |
| ＞10.3 | 43(7.65%) | 41(8.06%) | 2(3.77%) |  |
| Oxygen partial pressure / oxygen concentration ratio | 452.00(305.50-570.50) | 452.00(315.00-573.00) | 348.5 | 0.546 |
| Alveolar-arterial oxygen partial pressure difference | 30.55(12.53-57.70) | 30.50(12.10-57.70) | 57.70(57.70-57.70) | 0.386 |
| Arterial / alveolar oxygen partial pressure | 81.00(61.55-104.40) | 81.00(63.50-104.80) | 73.15 | 0.737 |
| Oxygen saturation,% | 97.80(96.00-99.00) | 97.80(96.00-99.00) | 95.00(82.40-) | 0.222 |
| ＜92.5 | 4(0.71%) | 3(0.59%) | 1(1.89%) | 0.053 |
| 92.5~98.5 | 51(9.07%) | 49(9.63%) | 2(3.77%) |  |
| ＞98.5 | 24(4.27%) | 24(4.72%) | 0 |  |
| Total hemoglobin, g/L | 19.10(14.15-113.50) | 20.20(13.50-112.50) | 15.70(15.00-131.50) | 0.780 |
| ＜120 | 52(9.25%) | 49(9.63%) | 3(5.66%) | 0.250 |
| 120~160 | 10(1.78%) | 8(1.57%) | 2(3.77%) |  |
| ＞160 | 4(0.71%) | 4(0.79%) | 0(0.00%) |  |
| **Imaging features** | | | | |
| Normal | 49(8.72%) | 42(8.25%) | 7(13.21%) | 0.454 |
| One side ground-glass opacity | 100(17.79%) | 93(18.27%) | 7(13.21%) |  |
| Both sides ground-glass opacity | 360(64.06%) | 326(64.05%) | 34(64.15%) |  |
| Consolidation | 12(2.14%) | 12(2.36%) | 0 |  |
| Others | 41(7.30%) | 36(7.07%) | 5(9.43%) |  |
| Data are median (IQR), n (%), or n/N (%). p values were calculated by Mann-Whitney U test, χ² test, or Fisher’s exact test, as appropriate. | | | | |
