# Supplementary material for: Clinical features and risk factors for severe inpatients with COVID-19: A retrospective study in China
Source: PLoS One. 2020 Dec 17;15(12):e0244125. doi: 10.1371/journal.pone.0244125 (PMC7745975; doi:10.1371/journal.pone.0244125)
Supplement: S4 Table — (DOCX) [file pone.0244125.s004.docx]

**S4 Table. Risk factors associated with disease severity for study patients in generalized linear model**

| **Parameter** | **Estimate** | **Standard error** | ***P* value** |
| --- | --- | --- | --- |
| intercept | 0.59397 | 0.10042 | <.0001 |
| age | 0.00392 | 0.0018 | 0.0318 |
| glucose | 0.19179 | 0.08471 | 0.0258 |
| Asthma | 0.15801 | 0.07211 | 0.0308 |
| fever | 0.08922 | 0.0513 | 0.0851 |
| White blood cell count | 0.03616 | 0.00861 | <.0001 |
